# “Covering provider”: an effort to streamline clinical communication chaos

**DOI:** 10.1093/jamiaopen/ooae057

**Published:** 2024-07-05

**Authors:** Mugdha Joshi, Arjun Gokhale, Stephen Ma, Anna Pendrey, Lauren Wozniak, Anoosha Moturu, Nicholas U Schwartz, Austin Wilson, Kelly Darmawan, Brian Phillips, Stav Cullum, Christopher Sharp, Gretchen Brown, Lisa Shieh, Clifford Schmiesing

**Affiliations:** Internal Medicine, Stanford University School of Medicine, Palo Alto, CA, United States; Clinical Informatics, Stanford University School of Medicine, Palo Alto, CA, United States; Clinical Informatics, Stanford University School of Medicine, Palo Alto, CA, United States; Geriatrics, Indiana University School of Medicine, Indianapolis, IN, United States; Adolescent Medicine, Stanford University School of Medicine, Palo Alto, CA, United States; General Surgery, Stanford University School of Medicine, Palo Alto, CA, United States; Neurology, Stanford University School of Medicine, Palo Alto, CA, United States; Nursing Innovation, Stanford Healthcare, Palo Alto, CA, United States; Stanford University School of Medicine, Palo Alto, CA, United States; Nursing Innovation, Stanford Healthcare, Palo Alto, CA, United States; Internal Medicine, Stanford University School of Medicine, Palo Alto, CA, United States; Internal Medicine, Stanford University School of Medicine, Palo Alto, CA, United States; Nursing Informatics, Stanford Healthcare, Palo Alto, CA, United States; Medicine, Stanford University School of Medicine, Palo Alto, CA, United States; Anesthesia, Stanford University School of Medicine, Palo Alto, CA, United States

**Keywords:** clinical communication, secure text messaging, provider assignment, quality improvement, lean

## Abstract

**Objective:**

This report describes a root cause analysis of incorrect provider assignments and a standardized workflow developed to improve the clarity and accuracy of provider assignments.

**Materials and Methods:**

A multidisciplinary working group involving housestaff was assembled. Key drivers were identified using value stream mapping and fishbone analysis. A report was developed to allow for the analysis of correct provider assignments. A standardized workflow was created and piloted with a single service line. Pre- and post-pilot surveys were administered to nursing staff and participating housestaff on the unit.

**Results:**

Four key drivers were identified. A standardized workflow was created with an exclusive treatment team role in Epic held by a single provider at any given time, with a corresponding patient list column displaying provider information for each patient. Pre- and post-survey responses report decreased confusion, decreased provider identification errors, and increased user satisfaction among RNs and residents with sustained uptake over time.

**Conclusion:**

This work demonstrates structured root cause analysis, notably engaging housestaff, to develop a standardized workflow for an understudied and growing problem. The development of tools and strategies to address the widespread burdens resulting from clinical communication failures is needed.

## Background

Delivering successful clinical care to today’s hospitalized patient requires significant time spent communicating between multiple members of a patient’s interdisciplinary care team.^1–4^ HIPAA-compliant secure text messaging applications are emerging to organize a patient’s care team around their complex and constantly developing care plan.^5–19^ Effective clinical communication is essential for high-quality care delivery and is an increasingly important domain within informatics.^20–22^ Communication failures are the largest contributor to adverse health outcomes, and ineffective communication in healthcare costs hospitals more than 12 billion dollars a year.^23–25^

**Figure 1.**
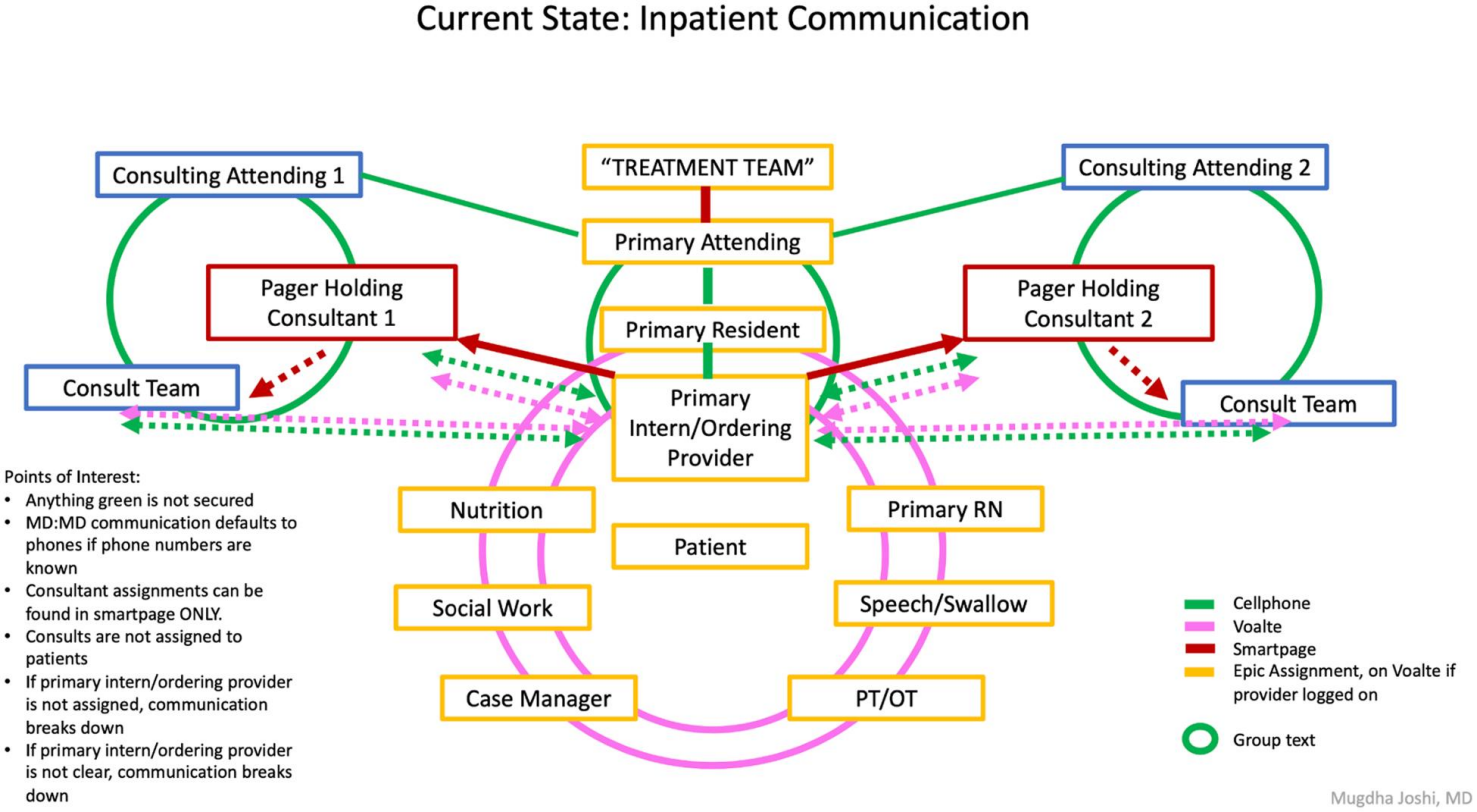
Graphic shows the organization of inpatient communication centering around the care of a hospitalized patient. The center depicts the primary physician team and all supporting services involved in the patients hospital stay. While much of the care coordination between members of the primary team and supporting services occurs via Voalté, much of the communication between physicians occurs outside with personal phone numbers. The primary message burden to the covering provider involves the Voalté messages from staff including RNs, Nutrition, Physical Therapy, Occupational Therapy, Speech Pathology, Social Work, and Case Management.

The care team is organized around the patient, with one provider sitting at the center of multiple lines of interprofessional communication (Figure 1). Every day at our hospital, over one hundred thousand clinical text messages are exchanged using our HIPAA-compliant text messaging application Voalté mobile, and the number keeps rising. Currently, 10 text messages are sent for every phone call. For the system to work reliably, for each of the thousands of messages initiated each day, it needs to be clear and straightforward to identify who the correct provider is responsible for a given patient’s needs.

This simple problem statement is complicated because secure text messaging has joined a complex ecosystem of existing workflows and telecommunication systems involving electronic medical records, paging systems, and personal mobile phones.^15^^,^^26^ Provider assignment information does not freely transmit between these different communication tools, and providers must be consistently accessible through multiple communication modalities simultaneously.^27^ In addition, the norms and practices of assigning oneself to a patient must be simple enough to be conducted reliably with each shift transition and quickly habit-forming for rotating trainees and traveling staff.^28^

Studies examining the transition to secure text messaging note several strengths, including fewer issues with “phone tag,” faster response times, greater team connectedness, and improved ease of communication between members of an interdisciplinary team.^15^^,^^19^^,^^29^^,^^30^ However, they also note an overwhelming increase in interruptions with consequences for the efficiency of care delivery, quality of care delivery, and quality of teaching at academic medical centers.^18^^,^^31–34^ Add to this burden of interruption the frustration of being unable to find the right person to contact or being incorrectly contacted in a slew of other interruptive messages, and the imperative to have a reliable and high-fidelity method of identifying the correct provider responsible for a patient becomes clear. A seemingly obvious ask: for every patient, it should be evident to every care team member who a patient’s responsible provider is and how to contact them.

## Objective

This study aims to describe root cause analysis, standardized workflow development, and implementation by a housestaff-led quality improvement committee to improve the ease and fidelity of identifying the correct first contact provider.

## Methods

### Organizational context

This intervention was developed and piloted at Stanford Healthcare (SHC), a quaternary care academic medical center with over 123 housestaff training programs, and both housestaff run and non-housestaff run services.

Pre Voalté implementation, clinicians communicated using a web-based paging system, Spok mobile (Spok, Inc, Alexandria, Virginia), with a personal cell phone or landline extension callback numbers. On admission, a patient would be assigned a “primary team” within SHC’s EMR system Epic (Epic Systems, Verona, Wisconsin). The name of this primary team and the associated pager number were visible from the patient’s EMR chart. In addition, each primary team had an associated “ghost” pager number which was forwarded to the individual pager for the covering provider. Nurses, therapists, and other clinicians paged the primary team pager to reach the person responsible for writing orders for a patient. At the end of the shift, the provider leaving the shift would forward their pager to a covering pager until they returned the next day.

Since Voalté was introduced in 2018, clinical service lines have developed heterogeneous workflows (Table 1). Providers assigned to patients in Epic can be messaged directly from Voalté when a patient is searched. During the assignment process, providers are prompted to provide their name and role (Primary Resident, Primary Intern, Attending, etc), displayed in Epic and Voalté. The roles used by different service lines are not standardized. Multiple providers with different roles are often assigned to a single patient, and nurses are expected to know which role is the right person to message. Additionally, every admitted patient continues to have a primary team assignment within Epic with the legacy primary team pager so the covering provider can be reached via pager. The result is a non-standard, redundant, and highly manual set of workflows prone to user confusion and communication errors. Providers report frequent incorrect contacts after the end of a shift or transfer of care. Nurses and other staff report significant time loss trying to identify the right person.

**Table 1.** Heterogeneity of provider assignment and communication workflows.

Time period	Scenario	Epic assignment	Voalté assignment	Intended RN contact behavior	Intended provider assignment behavior
Pre Voalté implementation	All patients	Primary team assignment	N/A	Primary team ghost pager	Team pager forwarded to oncoming provider
Post Voalté implementation	Patient 1: Voalté based workflow, all assignments correct	Primary team primary resident primary intern attending	Primary resident primary intern attending	Message primary intern	Cross-cover intern assigns with this role and must remember to unassign at end of shift Team pager forwarded to oncoming provider
	Patient 2: Voalté based workflow, cross-cover incorrectly remains assigned	Primary team primary resident primary intern attending Cross-cover intern	Primary resident primary intern attending Cross-cover intern	RN aware that cross cover should have logged off by this time in the day and messages primary intern 1	Cross-cover intern assigns with this role and must remember to unassign at end of shift Team pager forwarded to oncoming provider
	Patient 3: Voalté based workflow, patient transferred from another team	Primary team 1 Primary resident 1 Primary intern 1 Previous resident 1 Previous intern 1 Attending	Primary resident 1 Primary intern 1 Previous resident 1 Previous intern 1 Attending	RN looks at who is writing orders or looks up who is covering team pager and messages that person	Provider assignment after a patient transfer or rotation change and unassignment of previous providers
	Patient 4: Pager based workflow	Primary team Attending	None listed Provider not assigned or logged into Voalté	Pager holder via pager	Individual forwards coverage of ghost pager to oncoming provider
	Patient 5: Hybrid workflow	Primary team Attending	None listed Provider not assigned but logged in and searchable on Voalté	RN looks up who is holding the pager and finds that person in the Voalté directory RN pages pager holder via pager	Individual forwards coverage of ghost pager to oncoming provider Oncoming provider logs into Voalté without assigning to patient
Post intervention goal	All patients	Primary team Attending Any combination of provider assignments	Covering provider Additional assigned providers as needed per service line needs or in error	RN messages covering provider	Off-going provider assigns oncoming provider to “Covering Provider Role” and is automatically removed from that role. Individual forwards coverage of ghost pager to oncoming provider as needed

### Committee

This project is a joint endeavor between the Resident Safety Council, the Housestaff Information Technology Enhancement Council (HITEC), and a hospital-led task force focusing on Voalté improvement. An interdisciplinary group of housestaff representing internal medicine, general surgery, neurology, obstetrics/gynecology, adolescent medicine, geriatrics, and internal medicine trained informatics, worked closely with informatics leadership, the Voalté hospital taskforce, and a dedicated project manager.

### Root cause analysis

Lean A3 methodology was applied to design a solution.^35^ Value stream maps were developed, tracing all Voalté assignment and unassignment actions involved in a patient’s inpatient stay from admission to discharge for medical and surgical admissions. The interdisciplinary housestaff group including surgical and medical residents generated these value stream maps collaboratively. All transitions of care involved from admission to discharge were identified first. Then each anticipated Voalté assignment needed to facilitate this transition of care was listed. The group identified pain points for each transition. These pain points were analyzed for themes and consolidated into a fishbone diagram and key driver diagram. Intervenable factors contributing to each driver were identified and used to guide design of an intervention aimed at improving provider identification that could be implemented and evaluated in the time span of 1 academic year.

### Implementation

An initial feasibility pilot was run on a single unit and a single primary team consisting of 2 daytime surgery housestaff and 1-night float housestaff for 1 month. The internal medicine housestaff service was subsequently chosen for the large-scale pilot with a census of 156 because the service has patients geographically spread throughout the units in the hospital and already has a predominantly Voalté-based workflow. Nursing education was led by a nursing informatics champion and consisted primarily of emails and announcements at nursing huddles. The housestaff co-leads (internal medicine) led provider education. It consisted of presentations at teaching conferences leading up to the pilot start date, reminder emails, and timed reminder texts on the pilot roll-out date and with the first rotation switch.

### Survey development and collection

Pre- and post-surveys were developed and disseminated to both RN and housestaff users. Pre-pilot responses were received from 45/95 nursing staff who were requested to complete the survey, and post-pilot responses were received from 19/95 nursing staff. All nurses involved in the survey worked on med/surg acute care units. In addition, pre-pilot responses were received from 28/142 internal medicine residents, and post-pilot responses were received from 14/42 internal medicine residents who had rotated in the study period following implementation. All housestaff that participated in the study were working on inpatient acute care teams. Intensive care and consulting services were not included in this pilot.

### Analysis of intervention efficacy

Pre- and post-survey responses pertaining to metrics of accuracy of provider assignment, accuracy of contacts, and perception of negative care impacts were reported as Likert scores spanning from 1 (*never any occurrences*) to 5 (*multiple daily occurrences*). Survey responses pertaining to user satisfaction were reported as Likert scores spanning from 1 (*highly dissatisfied*) to 5 (*very satisfied*). Mann–Whitney U tests were used to determine statistical significance in changes in the median response pre and post intervention. This non-parametric test was chosen given its robustness to non-normal distributions and suitability for the ordinal nature of the Likert score data, with 2 comparator groups pre and post intervention, and unpaired observations. Statistical analysis was performed using Python with an alpha level set at 0.05 without correcting for multiple comparisons. Results of all statistical tests have been included. RN and resident responses were not compared to each other given the different interaction with the workflow between the 2 surveyed groups. Additionally, a daily report was generated within Epic depicting the number of patients using the Covering Provider role. The daily role use count was recorded and reported as a fraction of the daily census.

## Results

### Root cause analysis

**Figure 2.**
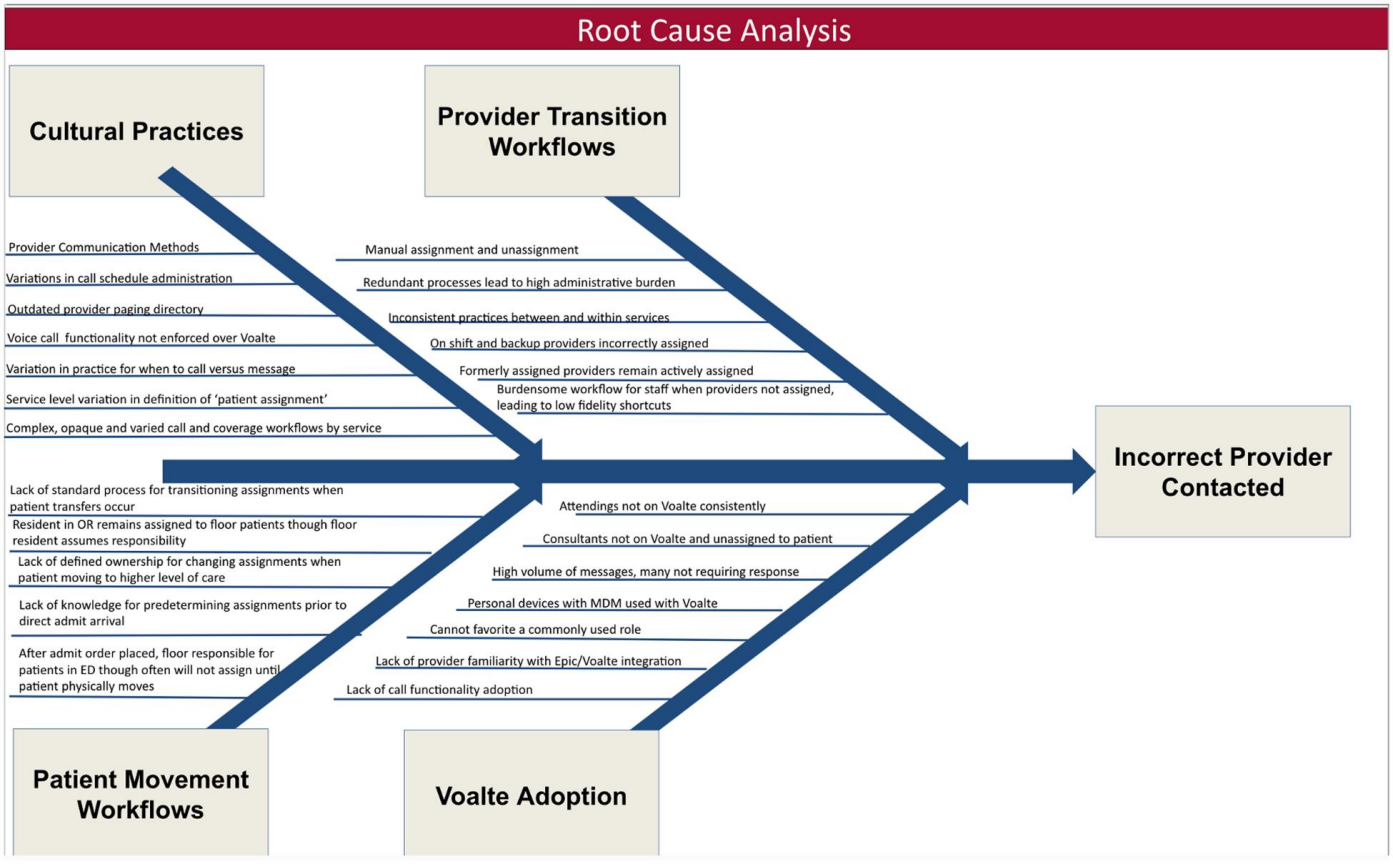
Fishbone diagram for the root cause analysis performed in this study organizing causative factors into 4 major domains contributing to incorrect providers being contacted.

The pain points identified from the analysis of the value stream maps (Supplementary figures) were organized into themes of cultural practices, provider transition workflows, patient movement workflows, and Voalté adoption. The fishbone diagram organizing the salient pain points by these themes is included in Figure 2. The themes highlighted set a broad institutional agenda for multi-pronged continued improvement efforts for Voalté usage. The key drivers of incorrect provider contacts were derived from these themes and intervenable factors from the fishbone diagram were used to guide development of an intervention of appropriate scope as follows:

*Driver 1:* The first contact provider must be clearly identifiable when correctly assigned.

The intervention should reduce the number of incorrectly assigned and formerly assigned providers remaining actively assigned and variation between definition of “patient assignment” between services should be minimized.

*Driver 2:* Provider transition workflows during shift change needs to be standard and reliable

The intervention should standardize expectations for provider assignment with sign out at shift change across service lines and all providers. The intervention should minimize manual steps of assignment and be simple and minimally burdensome to users.

*Driver 3:* Workflow for provider assignments during patient movement in the hospital needs to be standard and reliable

The intervention should standardize expectations for provider assignment with sign out at patient transitions of care across service lines and all providers. The intervention should minimize manual steps of assignment and be simple and minimally burdensome to users.

*Driver 4:* Voalté must be adopted and used by providers across the board.

The intervention should make Voalté more favorable to use by reducing incorrect message burden and increasing provider satisfaction.

### Intervention development

Using the drivers and contributing factors felt by the group to be most intervenable as a guide, the group developed a simple, standard workflow was developed with the goal of clearly identifying the first contact provider and could be reliably replicated at each transition of care. An exclusive assignment role was created within Epic called “Covering Provider,” which can be held by only one person at any given time. A corresponding patient list column was developed in Epic, which displays the name of the “Covering Provider” and the individual pager number. A standardized workflow was developed, which involved the expectation that a reassignment of the “Covering Provider” role accompanies every sign-out at shift change. Nurses were educated to message the person assigned as “Covering Provider” as first contact for patient care concerns.

To address driver 1, when correctly executed, the intervention standardizes the expected role name for the first contact provider and clearly labels the appropriate provider in Voalté and Epic. The “Covering Provider” column identifies the assigned provider and associated pager number from the patient list screen. The exclusive nature of the role eliminates formerly assigned individuals and allows for separate identification of backup providers who would need to have separate role assignments. To address drivers 2 and 3, development of a standard workflow creates specific expectations for assignment to a patient with handoffs of care. The exclusive functionality allows for an assignment with an automatic unassignment of the outgoing provider with a single step thereby reducing manual assignment burden and ensuring reliability. To address driver 4, the evaluation of the intervention was designed to focus on reduction of incorrect message burden and overall workflow satisfaction.

### Survey results

#### RN and resident survey results

**Figure 3.**
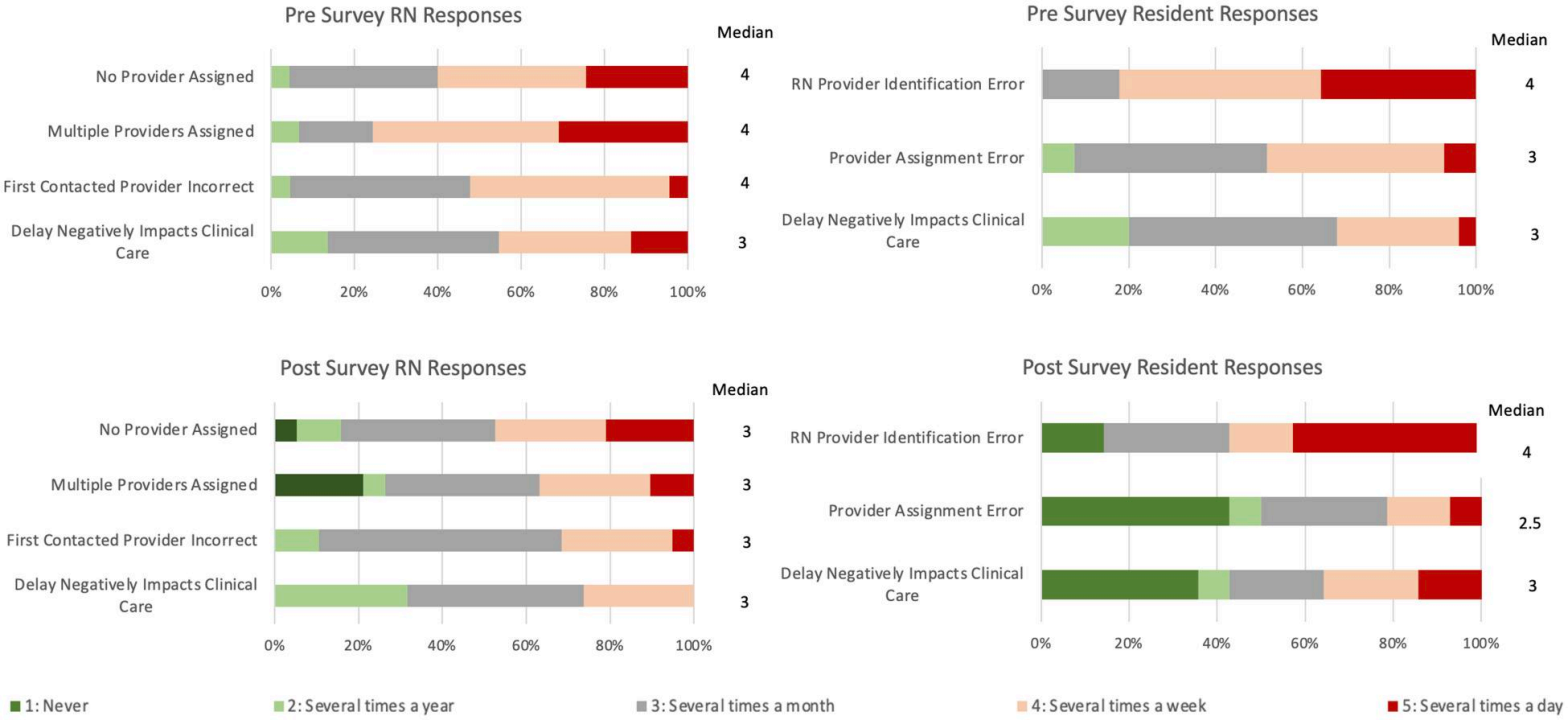
Each panel represents the distribution of responses for questions administered to RNs (left) and residents (right) pre (top) and post (bottom) intervention. Lower numbers, marked green, are favorable because they represent lower perceived frequency of negative events compared to the higher numbers marked red. Post panels (bottom) show a greater proportion of green and grey responses compared to the corresponding pre panel corresponding with the trend toward lower median responses.

RN and resident survey results for the metrics of interest are visually summarized in Figure 3 and change in medians reported in Table 2. Higher scores are undesirable as a score of 5 indicates multiple daily occurrences of errors in provider assignment, first contact provider contacted, and negative impacts in clinical care. RNs reported statistically significant decreased perception of incorrect first contact attempts with a decrease in median score of 4 (*several times a week*), to 3 (*several times a month*), *P* = .034 and statistically significant decreased perceived instances of multiple providers being assigned with a decrease in median score of 4 (*several times a week*), to 3 (*several times a month*), *P* = .003. While there was a decrease in median score for instances where no providers were assigned, this decrease was not statistically significant. There was no change in median score for perception of delays negatively impacting clinical care, however when viewing the visual distribution of responses, there appear to be a larger proportion of favorable (green) and neutral (grey) responses compared to unfavorable (red) responses in the RN post-survey compared to the pre survey. Taken together the responses indicate a trend toward improvement in errors in provider assignment, errors in first attempt at provider contact, and delays in care. Importantly, the intervention did not worsen any of these metrics.

**Table 2.** Change in median survey response stratified by respondent type on pre- and post-pilot surveys.

	RN		
Survey question	Pre (median)	Post (median)	*P* value
Delay negatively impacts clinical care	3	3	.034
First contacted provider incorrect	4	3	.144
Multiple providers assigned	4	3	.003
No provider assigned	4	3	.301
Satisfaction	3	4	.002

	Resident		
	Pre (median)	Post (median)	*P* value
Delay negatively impacts clinical care	3	3	.385
Provider assignment error	3	2.5	.009
RN provider identification error	4	4	.488
Satisfaction	2	4	.000

Residents reported statistically significant decreased perceived instances of being incorrectly contacted due to provider errors with logging out or unassigning themselves with a decrease in median score from 3 (*several times a month*) to 2.5 (*between several times a month and several times a year*), *P* = .009. While there was no decrease in median for messages being sent to the incorrect team member or perception of communication delays negatively impacting care, the visual depiction of the distribution of responses shows a greater proportion of favorable responses in the post pilot survey. Following the pilot, 14% of residents reported never receiving messages that should have been directed to other team members, 42% of residents reported never incorrectly receiving messages because of errors with unassigning or logging out, and 36% of residents reported there were never any communication delays that negatively impacted clinical care compared to 0% reporting “never” to these questions prior to the pilot. The resident responses also indicate overall improvement in errors leading to incorrect provider contact and again, the intervention did not worsen any of the metrics.

Post-survey free text quotes expressed interest in the idea with the feeling that further education for nursing and other support staff was needed to reduce the number of incorrect contacts.

### Overall satisfaction

**Figure 4.**
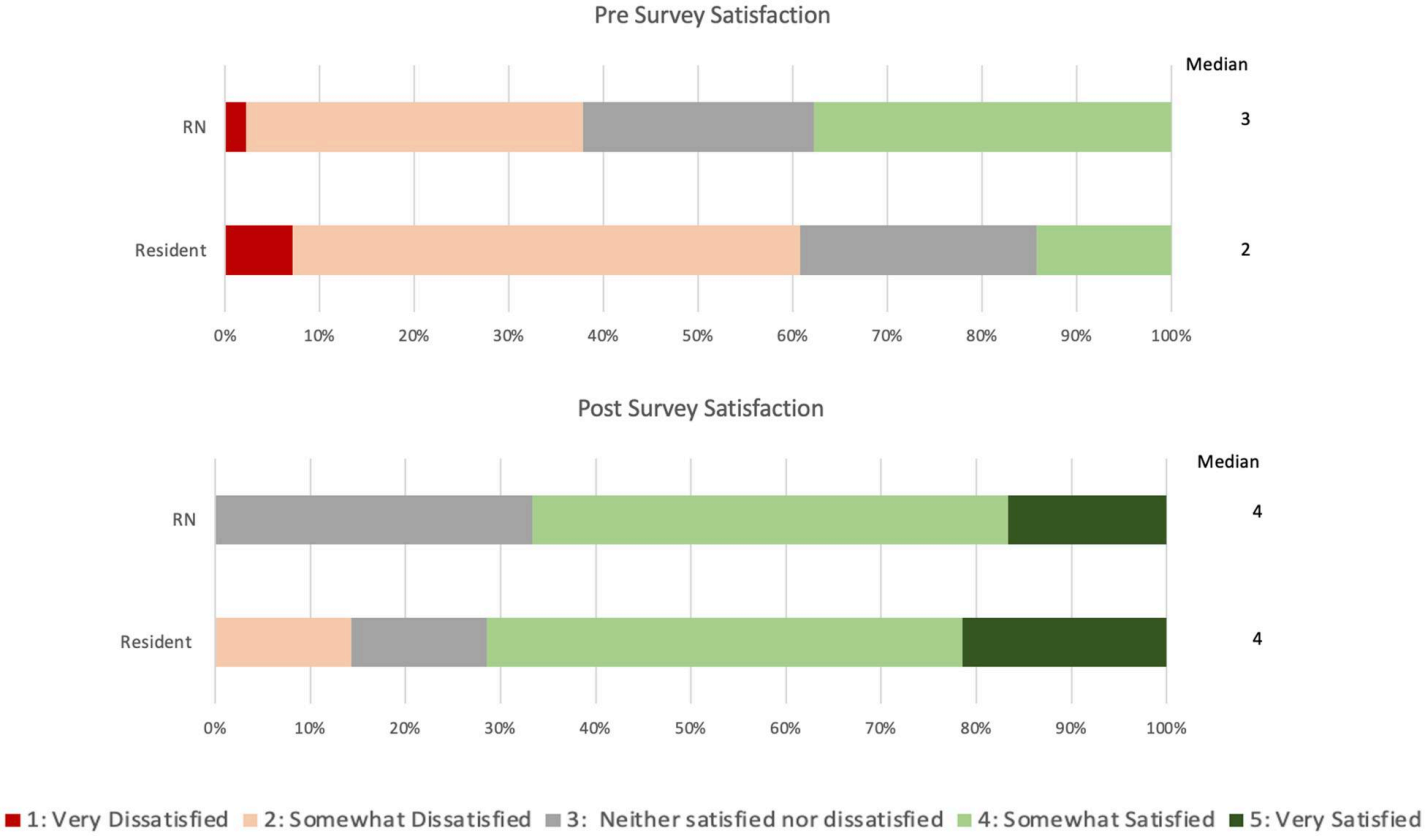
Top panel represents pre-survey workflow and bottom panel represents post-survey workflow satisfaction stratified by respondent type (RN vs resident). Higher numbers, marked green, are favorable because they represent higher satisfaction compared to the lower numbers marked red. Post panel (bottom) shows a greater proportion of green responses compared to the pre panel corresponding with the higher median responses.

Satisfaction results are summarized in Figure 4 with changes in median scores reported in Table 2. Higher scores are desirable as a score of 5 represents a response of very satisfied. Both RNs and residents reported a statistically significant overall increase in satisfaction with the intervention workflow. RN median satisfaction increased from 3 (*neither satisfied nor dissatisfied*) to 4 (*somewhat satisfied*), *P* = .002. Resident median satisfaction score increased from 2 (*somewhat dissatisfied*) to 4 (*somewhat satisfied*), *P* = .000. Moreover, only 38% of RNs reported being somewhat satisfied, and 0% reported being very satisfied with the communication workflow before the pilot, compared to 67% of RNs reporting satisfaction after the pilot, with 17% reporting being very satisfied. Similarly, only 14% of residents reported being somewhat satisfied, with none reporting being very satisfied prior to the pilot. Resident satisfaction increased to 71% post-pilot, with 21% very satisfied.

### Intervention uptake overtime

**Figure 5.**
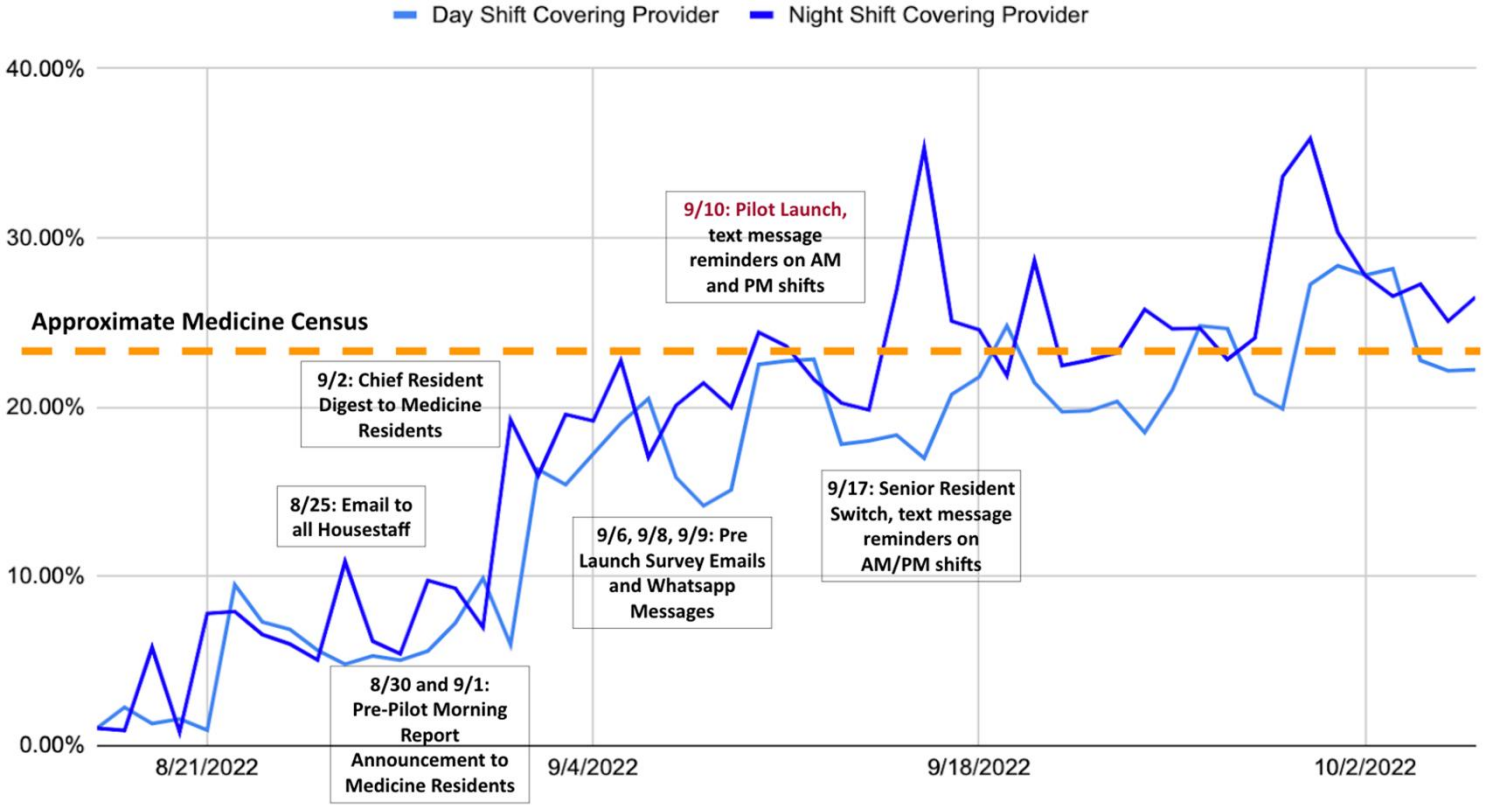
Plot shows percentage of patients with covering provider role assigned out of the total hospital census over time. The dotted line represents the approximate medicine census calculated as the total number of patients allowed on all housestaff medicine services (156) out of the average hospital census (686). The different outreach efforts to encourage the intervention uptake are shown.

Use of the role steadily increased with intervention reminders in the days leading up to the pilot. The role remained in use even after reminders for use stopped and has maintained itself without reminders for months. Night shift providers were more consistently assigned using the covering provider role than daytime providers (Figure 5).

## Discussion

This analysis outlines the multifactorial contributors to the difficulty of correctly identifying a patient's care provider. It applies Lean A3 methodology to develop a streamlined, context-specific solution to a common communication problem encountered across hospital systems.^35^ The intervention demonstrated improvement across all surveyed measures for both providers and nurses, particularly with regards to ambiguity from provider assignment errors. Importantly, none of the surveyed measures appear to have been negatively impacted by the intervention. Most remarkably, the workflow improved overall satisfaction with the state of clinical communication among all users. It also demonstrated persistent uptake over time with minimal user education.

Difficulty with provider identification is familiar and was present and understudied even in the pager era. In 2009, one study reported that 14% of pages were sent to the wrong physician while the physician was post-call, in the evening, or during protected academic time. A review of the text showed that 15% of these were emergencies, and 32% needed a response in an hour. These results were consistent across 2 teaching hospitals with different call schedules and paging systems, supporting the importance of this issue we describe at our institution.^36^ At a high level, challenges with updated physician contact information, redundancy from multiple electronic systems that do not integrate, and unforeseen consequences from implementing new technology in a complex existing technological infrastructure have been reported before.^37^ Some published support imploring for more role-based contact to help reduce confusion with changing shifts, and anecdotally solutions similar to ours have been developed in parallel at other institutions.^38^ However, this is the first published report we are aware of describing any intervention addressing this problem.

The results of this analysis are subject to non-response bias as rates were low for both nurses and residents before and after the pilot. Those who responded prior to the pilot may have had more grievances with the prior workflow, and those who responded afterward may have been more eager to support the ongoing use of the new workflow. Additionally, the statistical analyses are limited by the fact that some of the respondents may have responded to both the pre- and the post-survey but were anonymous and therefore unable to be paired for analysis. However, ongoing use by providers without additional reminders suggests ongoing acceptance, which may be motivated by the overall satisfaction increase reported in the survey. Furthermore, response changes were congruent in that nurses reported increased ease of identifying the correct person, and residents reported decreased contact errors.

A major facilitator for this intervention's success was the time spent on a deep root cause analysis and solution derivation from understanding the many contributing factors to this problem. In addition, the sustained uptake of the workflow with minimal instruction supports that it is a simple and intuitive workflow, serving as a case study for applying a lean methodology to a complex, unruly healthcare problem. The effort was also significantly facilitated by the direct engagement of nursing and resident stakeholders in the solution's design and the intervention's rollout, particularly involvement of internal medicine housestaff in the development and rollout of an intervention piloted with the internal medicine service. Engagement of housestaff quality and informatics interest groups was a strength of our approach and supports the idea expressed by others that engagement of housestaff in informatics initiatives is an opportunity to develop more sustainable and effective workflows.^39–41^ Furthermore, the interdisciplinary nature of the groups involved, with representatives who work in medical and surgical service lines and hold both consulting and primary roles, led to a deeper root cause analysis and development of a more universally applicable solution with champions across departments.

A significant challenge to the success of this intervention is the geographical spread within the hospital of the different users. Educating nurses across different units was difficult. Furthermore, other care team members, such as physical therapists, occupational therapy, and case managers, could not be included in direct education efforts and therefore were not included in the surveyed individuals. Providers continued to receive messages directed to incorrect care team members from these non-nursing members of the care team and nurses who were missed by educational efforts. Similarly, nurses continued to have patients whose providers followed different assignment workflows since the pilot was limited to one service line. This diluted the designed simplicity of the intervention by continuing to require nurses to switch between workflows for different patients they may be caring for. As noted by others, consistency of a communication workflow across a hospital system is critical for success.^37^ The hope is to use this pilot's feasibility and overall positive feedback to increase buy-in for use in service lines as we roll out across the hospital.

## Conclusion

Dissatisfaction with clinical messaging systems due to untenable message burden and confusing provider assignments is a growing problem nationwide, with limited reporting of solutions to date. In addition, the problem is so heterogeneous and multifactorial that addressing it within one hospital requires a large interdisciplinary undertaking. Lean A3 methodology can be applied to develop a context-specific solution to identify the responsible patient care provider correctly. Creating a role that can be held by only one provider and creating clear expectations around role reassignment at transitions of care can lead to decreased confusion, messaging mistakes, perceptions of delayed care, and increased overall satisfaction with sustained uptake over time. Further study and best practices around cleaning up the chaos of clinical communication are needed to successfully use secure text messaging applications in a way that supports patient safety and workplace wellness.

## Supplementary Material

ooae057_Supplementary_Data

## Data Availability

The data are available to be shared upon reasonable request to the corresponding author.
