# Supplementary material for: “Covering provider”: an effort to streamline clinical communication chaos
Source: JAMIA Open. 2024 Jul 5;7(3):ooae057. doi: 10.1093/jamiaopen/ooae057 (PMC11226879; doi:10.1093/jamiaopen/ooae057)
Supplement: ooae057_Supplementary_Data [file ooae057_supplementary_data.pdf]

VSM: Provider Assignments – Medicine Service: Floor w/ Procedure

Stanford HEALTH CARE

Stanford MEDICINE | Resident Safety Council
Quality Improvement & Innovation

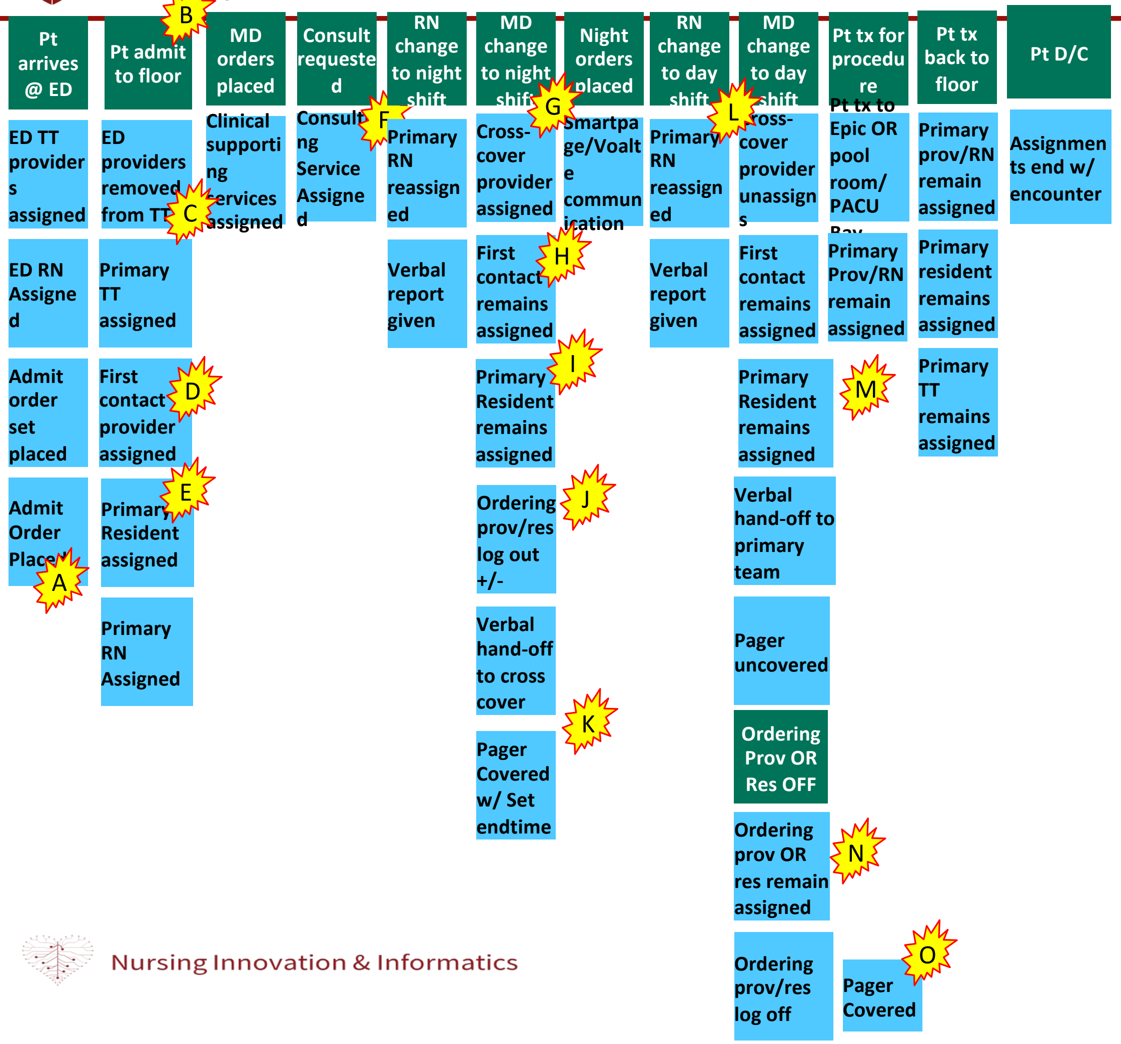

Pain Points:

A.Contacting attending to accept patient

- Method and medium to contact attending
  - Not 100% on Voalte
  - Not 100% on SmartPage
  - Usually ED will page the resident on the service who has to figure this out

B. Pt direct admit – can arrive on floor with only Attending assigned

C.“Qualifying ADT message” for time-based assignment do not update Voalte care team automatically

- Manual assignments qualify, and update care team

D/F Needs to be a manual assignment, not always assigned, different titles on different services

E/I. Primary resident not always assigned, primary resident may not be expected to write orders, may be off

G/H. Cross-cover provider assignments not consistently made

F. Consulting Provider not assigned to patient

- Smart Page for Consulting Service does not always display named user
- Consulting team usually does not assign themselves to patient, just “log on” to Voalte and are searchable

- Pt tx to OR pool room (off-unit)
  - No pt integration with Epic Optime; no MD assignment updates made
  - Pt tx to Imaging will stay in same pt room/bed in Epic/Voalte

L. Crosscover can add end time to Epic assignment which makes unassignment automatic, not consistently done. Sometimes manual and crosscover remains assigned

H/I/L/M/N: No culture of unassigning ordering providers/residents at end of shift or on days off from Epic

J. Providers forget to log off on Voalte when they leave so they appear to still be assigned even if at home

K/O. Calling to cover pagers is a manual and time consuming process. Introduces delays in assignment to patients because cross cover waits to have collected a few pages prior to calling.

- Person covering pager calls operator to assign to specific pagers for certain period of time
- If pager doesn’t get covered, often found out because person holding pager gets paged at home

Nursing Innovation & Informatics

VSM: Provider Assignments – Medicine Service: Floor to ICU

Pt tx to ICU	RN change of shift	MD change of shift	Pt tx back to floor	Pt D/C
A MICU TT assigned	Primary RN reassigned	C Physical transfer of team phone	Primary TT reassigned	Assignments end w/ encounter
MICU provider may assign +/-	Verbal report given	Verbal report given	MICU provider may assign +/-	
Pt physically moves			New Unit RN assigned	
New Unit RN assigned			Level of care order placed	
B Prior team members removed				
Prior RN removed				

Pain Points: (Where can we get data to support this?)

A. Primary team members not consistently unassigned

B. On transfer, teams will intentionally want to remain assigned as primary team in order to follow patient (Onc APP service Med 9/11). Minor issue as patient is in ICU based on room number and people know who to contact

C. All messages w/ ICU team phone go to single person holding phone even though multiple providers are on the team

- If MICU provider has personally assigned, inconsistent unassignment when patient sent back to floor

VSM: Provider Assignments – Medicine Service: Change in Floor Primary Team

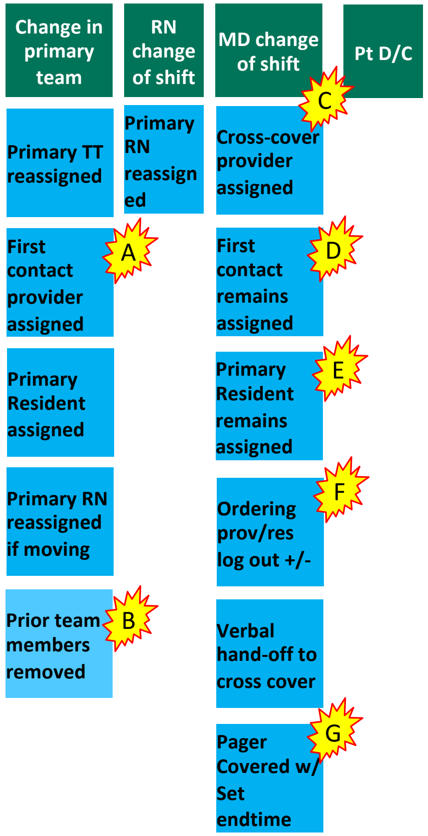

Pain Points:
A. New first contact manually assigned which offers opportunity to miss it
B. Prior team members often remain assigned
C-F: Can lead to situation where prior first contact, new first contact, and cross cover are all simultaneously assigned

- A. Process for whether TT alone, TT + first contact is assigned varies depending on new primary service
- All prior slide pain points apply

Stanford HEALTH CARE

VSM: Provider Assignments – Surgical Service

Stanford MEDICINE | Resident Safety Council
Quality Improvement & Innovation

Pt arrives @ ED
- ED TT providers assigned
- ED RN Assigned
- Paged via Spok by ED secretary
- Admit Order Placed
- Admit order set placed
- Consulting resident no longer responsible for floor pa[illegible] (1)

Pt admit to AAU unit
- ED providers removed from TT
- Primary service assigned (3)
- First contact provider assigned
- Primary RN Assigned

Direct Admission to Floor (2)
- Attending assigned automatic
- Floor nurse assigned, sends page to resident to assign and [illegible]

RN change of shift
- Primary RN reassigned

Pt in procedure/OR (4)
- Voalte use in OR spotty
- Limited" communicatethrough [illegible]
- No integration of OR staff

MD change of shift/night float (5)
- Cross-cover provider assigned – currently not used
- General Surgey developed night flaot Voalte assignments "Triple T's" and "Night Tens"

MD orders placed
- Clinical supporting services assigned

Consult requested (6)
- Consulting Service Assigned

Pt tx for procedure
- Pt tx to Epic OR pool room/ PACU
- Pt tx to Epic OR pool room/ PACU Bay

Patient in PACU (7)
- Voalte currently not used in PACU - trained in Spok

Pt tx back to AAU
- Prior team resumed

Pt tx to ICU (8)
- Primary ICU service reassigned

Change in primary team
- Primary TT reassigned

RN change of shift
- Primary RN reassigned

MD change of shift
- Hand-off to Resident

Pt tx back to AAU
- Primary service reassigned

Change in TT
- Primary TT reassigned

Pt D/C

Pain Points: (Where can we get data to support this?)

1. ED consult
   - ED Team not always assigned – unclear during shift changes – originally page is via spok and requires call back (time consuming)
   - Contacting attending to accept pt – most services contact own service attendings via cell – not actionable
   - After completion of consult resident defers care to intern for floor team – not always signed out well
2. Pt direct admit – can arrive on floor with only Attending assigned
   - ALLOW FOR AUTOMATIC ADDITION OF TEAM (always the same)
   - Ideally automatic addition of resident (would require resident assigned to treatment team in epic
3. Old providers staying on chart after transfer
   Allow for reset of assignments on transfers / can manually remove other providers

- 4. See OR pain points slide – not viable in OR as privacy – nurse must open personal phone, connection issues, OR staff not assigned – **possible fix point would be nurse to nurse communication when scrubbed**
- **5.** "Qualifing ADT message" for time-based assignment do not update Voalte care team automatically
  - Manual assignments qualify, and update care team - – many night float default to spok, home call if difficult to wake up to voalte notifications.
- 6. Cross-cover provider assignments not consistently made
- Consulting Provider not assigned to TT, only service
  - Smart Page for Consulting Service does not always display named user
- Pt tx to OR pool room (off-unit)
  - No pt integration with Epic Optime; no MD assignment updates made
  - Pt tx to Imaging will stay in same pt room/bed in Epic/Voalte

7. Pt in PACU
   - PACU staff currently only using Spok – which is covered by intern who generally not aware of surgical course
8. Same theme. ICU team generally does not assign directly under patient (uses triage or bat phone)

Nursing Innovation & Informatics

VSM: Provider Assignments – Consultant

- **Pt arrives @ ED**
  - ED TT providers assigned
  - ED RN Assigned
  - Paged via Spok by ED secretary
  - Admit Order Placed
  - Admit order set placed
  - Consulting resident no longer responsible for floor pages [1]
- **Pt admit to AAU unit**
  - ED providers removed from TT [2]
  - Primary service assigned
  - First contact provider assigned
  - Primary RN Assigned
- **Direct Admission to Floor** [3]
  - Attending assigned automatic
  - Floor nurse assigned, sends page to resident to assign and place orders
- **RN change of shift**
  - Primary RN reassigned
- **Pt in procedure /OR** [4]
  - Voalte use in OR spotty
  - Limited"communicat ethrough Epic
  - No integration of OR staff
- **MD change of shift/night float** [5]
  - Cross-cover provider assigned – currently not used
  - General Surgery developed night float Voalte assignments "Triple T's" and "Night Tens"
- **MD orders placed**
  - Clinical supporting services assigned
- **Consult requested** [6]
  - Consulting Service Assigned
- **Pt tx for procedure**
  - Pt tx to Epic OR pool room/ PACU Bay
  - Pt tx to Epic OR pool room/ PACU Bay
- **Patient in PACU** [7]
  - Voalte currently not used in PACU - trained in Spok
- **Pt tx back to AAU**
  - Prior team resumed
- **Pt tx to ICU** [8]
  - Primary ICU service reassigned
- **Change in primary team**
  - Primary TT reassigned
- **RN change of shift**
  - Primary RN reassigned
- **MD change of shift**
  - Hand-off to Resident
- **Pt tx back to AAU**
  - Primary service reassigned
- **Change in TT**
  - Primary TT reassigned
- **Pt D/C**

1.In the ED: Difficulty identifying the person assigned to the patient, for f/u of recommendations and plan of care.
Consultants don't want to add themselves due to excessive messages better served to primary team.
2.In the Unit: If consult placed in ED; provider from ED is still assigned when patient arrives to the floor.
- Often caught in large group messages that aren't pertinent and difficult to take off easily
- Difficult to get patient name in subject line for 1:1 message
- First and second call system is not established
3.Direct Admit: Difficult for consultant team to know who is responsible for patient to discuss plan
4. Unclear often who is primary especially with multiple surgical sub-specialties.
5. Cross cover provider assignments not consistently made. Takes a lot of time to add yourself as a cross cover, not easy to highlight everyone as a cross cover, people don't always do that.
- Not easy to take yourself off a patient
6. CDU APPs and attendings often are not assigned, and there are often acute issues with these patients.

1-8
-Difficult for consultant team to know who is responsible for patient to discuss plan.
-Two systems to communicate and to consult providers: Voalte and Pager: Inconsistency of providers signing to Voalte.
- High priority messages underutilized.

Pain Points in OR/procedure rooms

Pain Points in OR/procedure rooms

- IP clinical teams use of Voalte spotty
- Attending surgeons do not use Voate
- Voalte app on personal phone in OR problematic
  - Unable to access on mobile phone while scrubbed – privacy issues when nurse open personal phones
  - Traditional pager better "form factor": no lock screen or PW issues
  - Resident in "first contact" role may be in OR, especially at night – with no backup to page
  - Hard to route or refer pages and text to another team member – can only self assign and unassign
  - Pagers can be "pushed" to another person
- Challenges/barriers for Preop and PACU staff to find care team member (1st contact) in Voalte for surgical patients
- Residents often leave a pager ID for PACU staff as point of contact, not Voalte
- Patient-centered integration on the IP does not work for perioperative patients. No integration with Epic – none.
- 100% self assignment and un-assignment for all on the IP.
- Somewhat complex and custom set up for OR teams and groups
- Incomplete active directory – hit or miss if you will find the person or the role you are looking for .
- Technical limitation: cannot "favorite" a role and cannot easily preconfigure common and frequently called IP phone numbers and roles.

Common and shared challenges and limitations of Voalte and clinical communication in general across workflows

Stanford MEDICINE | Resident Safety Council
Quality Improvement & Innovation

- Requirement to self assign and unassign in Voalte and Epic and Smartpage
- Persistence of Smartpage/Spok for clinical communications
- Large volume of clinical/patient-specific team-based communication through personal cell phone text and call, outside of Epic, Smartpage/Spok, and Voalte
- Need to manage roles and availability manually and simultaneously in multiple systems: Epic, Smartpage, and Voalte
- Complex, non-standard, and non-transparent call and coverage workflows across clinical teams and services
- No enterprise scheduling system
- Challenges/impossibility of knowing the right person to reach (primary team 1st. contact, consult service, admitting clinician, triage, cross coverage)
- Partial adoption of Voalte among clinicians, especially attending physicians
- Limited role of Voalte for clinical/ambulatory areas
- interruptive and excessive texting and clinical communication affecting clinical effective and clinician wellness
- Lack of data, metrics, and analytics guiding use of these complex and novel workflows and platforms.
